# Evaluation of Facilitators and Barriers to Implementing Evidence-Based Practice in the Health Services: A Systematic Review

**DOI:** 10.31661/gmj.v9i0.1645

**Published:** 2020-03-14

**Authors:** Ali Ayoubian, Amir Ashkan Nasiripour, Seyed Jamaledin Tabibi, Mohammadkarim Bahadori

**Affiliations:** ^1^Department of Health Services Management, Faculty of Medical Sciences and Technologies, Science and Research Branch, Islamic Azad University, Tehran, Iran; ^2^Health Management Research Center, Baqiyatallah University of Medical Sciences, Tehran, Iran

**Keywords:** Barriers, Facilitators, Evidence-Based Practice

## Abstract

**Background:**

Evidence-based practice (EBP) is an ambition for health service administrators. We aimed to systematically review the major relevant articles in case of barriers and facilitators to implementing evidence-based practice in health services.

**Materials and Methods:**

The type of study was a systematic review. We searched the libraries and online sources such as PubMed, MEDLINE, Wiley, EMBASE, ISI Web of Knowledge, Scopus, Science Direct, Cochrane Library, and Google scholar. We used keywords included "Evidence-Based Practice", "Evidence-Based Management", "Healthcare", "Care Management, Evidence-Based Healthcare Management", "Health Care", Health", "Barrier", "Facilitator", policy and "Evidence-Based Healthcare".

**Results:**

In total, 12 studies were included. Several barriers and facilitators were recognized through the included papers, the factors such as organization support and a helpful education system improved skills, knowledge, and confidence to EBP. The outcomes of studies were identified as the employ of the internet as a highest-rated skill for increasing EBP quality.

**Conclusion:**

Generally, the results showed health service administrators should first identify barriers of EBP then transferred them to facilitators to the implementation of proper and efficient EBP.

## Introduction

Evidence-based practice (EBP) is an ambition for health service administrators. EBP has been the combination of best study evidence with clinical expertise and patient uses in the decision-making method for patient administration [1-4]. EBP is an essential function in upholding the national health system; it supports efficient interventions and therefore presents the ground for evidence-based administration of supplies and workforce. Recently there has raised interest, comprehensive, in evidence-based health policy and interpretation of study to work [5,6].

Many eastern Mediterranean countries are lagging behind in biomedical study publications and interpretation of study evidence into health policy and programs, the applications have been restricted in developing such capability [6-8]. One of the principal barriers to the implementation of EBP is the shortage of science and abilities required for the interpretation of evidence into applications and policy [8-10]. EBP has been slowly utilized by healthcare workers in the globe [7,8]. So, there is a noticeable gap between this perfect situation and the status quo [6-10]. The major factors were identified from the different studies. EBP has been influenced by various factors such as the organizational, strategies, individual and social circumstances. But, dependent on participants’ field and different studies, various facilitators and barriers to implementing EBP in the health services have been reported [1,2,10,12]. In this study, we proposed to systematically review of the major relevant articles in case of facilitators and barriers to implementing EBP in the health services in order to help clinical managers and administrations to have a better knowledge of these technologies and deciding the better therapeutic decision.

## Search Strategies

**Figure 1:**
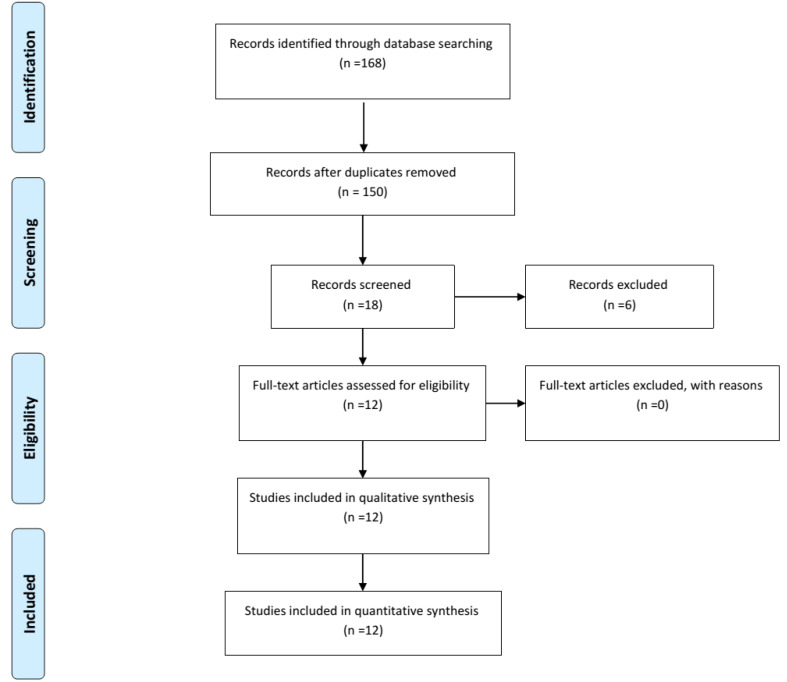
The PRISMA flow chart depicts the database explorations, abstracts selected and the papers included

 We searched the libraries and online sources such as PubMed, MEDLINE, Wiley, EMBASE, ISI Web of Knowledge, Scopus, Science Direct, Cochrane Library, and Google scholar. We used keywords included “Evidence-Based Practice”, “Evidence-Based Management”, “Healthcare”, “Care Management”, “Evidence-Based Healthcare Management”, “Health Care”, “Health”, “Barrier”, “Facilitator”, “Policy and Evidence-Based Healthcare.” Two reviewers AA and AAN were independently evaluated the titles and abstracts of all studies. The conflicts were solved by the third reviewer (LN). The two reviewers AA and AAN who conducted the literature search also individually made decisions and selected studies. The differences were solved by either discussing a third reviewer (LN) or argument. For methodological quality before inclusion papers in the review, the CASP checklist for qualitative research was used. The CASP checklist was used from 10 questions with 3 potential responses: “yes”, “no” and “can’t tell” to evaluate qualitative research. If greater than eight of the questions on the questionnaire were fitted, the research was assessed as good quality; if five to seven were fitted, it was assessed as fair quality; and if less than five were fitted, it was marked as poor quality. Figure-1 showed a Prisma flow chart for this study.

## Results

 Our search initially retrieved 168 studies published up to January 2019. However, 150 articles were removed for the reason duplication between databases. Then, 18 papers were involved for initial selection. Upon screening abstracts and titles, 12 papers were recognized for full-text review (Figure-1). Table-1 showed studies’ characteristics regarding the author’s name (data), sample, design, purpose, barriers, and facilitators. The quality of the studies varied between five to eight out of 10 questions (fair-to-good quality).

###  Barriers and Facilitators 

In the nurses, barriers constituted a shortage of accessible data in Chinese, nurses’ absence of knowledge of what EBP indicates, and worry that patients will be offended of obtaining overhaul that is regarded as modern. Facilitators involved administration support and the fast grown of social network services [1]. The most common barriers in the nursing homes were including difficulty understanding statistical analyses, the inadequate authority to change practice and perceived isolation from knowledgeable colleagues with whom to discuss the research. Also, the facilitators were efficient research training, accessibility to Internet facilities and improved computers, and collaboration with academic nurses [8]. The greatest barriers in psychiatric nurses were insufficient resources to modify practice and insufficient time to find and read research reports. The most supportive resource for altering was reported as practice development coordinators. The highest-rated skill was using the Internet to search, and the lowest-rated skill was using research to modify practice for developing evidence-based practice [9]. The absence of skills and knowledge, restricted support, poor time allowance, and inadequate resources are as barriers to acceptance of EBP in health care for nurses employed in a tertiary health care network [13]. Also, another study related to the nurses discovered that the blocks to education EBP were the absence of initial investment, skill, and knowledge for teaching EBP; rules-oriented nursing culture, hierarchical; limited research application and dissemination, latent student overloads in treating EBP. Facilitators were recognized as the collaboration in hospitals and schools; the importance of EBP to the work of nursing; and ongoing teaching in teaching/utilizing EBP [4]. The majority of barriers related to geriatric, psychiatric were including “Insufficient resources for applying investigation results and “Inadequate training in research methods”. The greatest facilitators reported were as follows: “Training in research methods” and “Organizational policies and protocols that are evidence-based” [2]. Six fields among pediatric surgeons were recognized as significant to altering pediatric surgeons’ usage of evidence in practice; goals, skills, knowledge, environmental resources and context, social/professional role and identity, and social influence. The main barriers to evidence-based practice employment included resource limitations and time constraints, a lack of required skills, the commonly poor quality of data in pediatric surgery, and a belief that remains to trust on a training style of education. Facilitators were as follows having a native peer/champion that supports EBP employment, and working in a research hospital [11]. The obstructions to evidence-based decision making (EBDM) in Iran’s health system classify into the research system, decision-makers’ characteristics, and the decision-making atmosphere, with each type including further related themes and subthemes. Also, there are multi-dimensional solutions that can strange the impact of scientific evidence on decision-making. Numerous documented obstructions to EBDM are fixed in health system stewardship, such as the ill-defined priorities and weakness of inter-sectoral collaborations [5]. In territorial chronic disease practitioners, organizational barriers have higher scores than personal barriers. The largest reported barriers to EBDM were inadequate funding, lack of incentives/rewards, a perception of state legislators not supporting evidence-based policies and interventions, and feeling the require to be a professional on many issues. In adjusted models, the women were more prone to the description of a lack of skills in communicating with policymakers and in developing evidence-based programs. Participants with a bachelor’s degree were more prone than those with public health master’s degrees to description deficient of skills in developing evidence-based programs. In contrast, the specialists, Men, and participants with doctoral degrees were all more prone to feel require to be a professional on many issues to efficiently make evidence-based decisions [6]. The analysis of data on behavioral professionals showed seven themes to explain both facilitators and barriers: (1) attitudes, (2) training, (3) logistical considerations, (4) consumer demand, (5) policy, (6) institutional support, and (7) evidence. The lacks of training and negative attitudes about EBP were most frequently barriers. Also, the growing evidence base is a major facilitator [7]. The main barrier and facilitator evidence-based behavioral sleep-related car associated with training, knowledge, and education. The facilitators included beliefs and supportive sleep attitudes, and the barriers included require institutional support and time [10]. Facilitators and barriers for EBP inpatient care consist of five scopes: subjective norm, decision making, perceived behavior control, attitude, and behavior and intention [12].

## Discussion

In general, different groups of health care providers have less knowledge about proprietary evidence-based terminology, and reference books are the most important source of information for EBP. The key concepts in EBP include scientific and professional care, patient-centeredness, and attention to the quality of service. The factors such as organization support and a helpful education system improved skills, knowledge, and confidence to EBP [14-17]. The studies demonstrate that the organization as a sustaining body for research-correlated actions but not for skilling the body to approve EBP. The enormous majority of participants in the previous studies robustly experienced that they required educational alteration to improve their examination and assessment skills [15-17]. Lack of adequate facilities, time, knowledge with the research method, and lack of discretion to make changes are the most important barriers to EBP; also providing adequate opportunity, training in research methodology, and holding evidence-based training courses as important facilitators have been suggested. The teaching of the principles of the research methodology and how to apply their results, conducting evidence-based performance training courses, regular, transparent and understandable information gathering in the organization are as the most important facilitators of EBP. These results are also consistent with the results of most studies [37-39]. Education can be used as a procedure to overcome EBP barriers and to produce positive approaches. Several studies reported that sufficient resources, organizational support, and use of verified educational policies are the main apparatus for the success of EBP [4-8,18-22]. The results of interventions in the field of EBP indicate a significant impact of these interventions. By examining and considering the most important barriers to EBP mentioned in the studies, we will find that these results are consistent with the results of many studies. Pagoto *et al*.reported that negative attitudes and lack of training were as the most important barriers [35] also, Bayley *et al* showed that time shortage was the main barrier to EBP [36]. The level of supposed barriers and the commonly cited barriers to using research has been consistent in current studies. However, the most repeatedly cited barriers differ among countries such as Africa, Iran, Australia, the USA, Sweden, and the UK [10,13,22-24]. The barriers of EBDM classified into three groups including research system, decision-maker characteristics, and decision-making environment. The key point is that the logical and proper connection must to be upholding between these three components to ensure a perfect EBDM process. If there is no effective and correct connection between these three domains. Innvaer *et al*. and Mitton *et al*. evaluated the obstructions to EBDM in health management and policymaking. They divided the obstructions of EBDM as follows: organizational level (incentives stronger than EBDM, non-supportive culture, unsuitable reward systems for researchers, rapid replacement of staff and absence of political stability), personal level (poor ability for studying and operating evidence and lack of experienced personnel, negative feelings toward change and absence of mutual trust), time-related factors (limited time for decision making and diverse time-frame), communication factors (high volume of data, poor selection of messenger, absence of an actionable message, inappropriate scientific language for policy-makers, and lack of direct communication), low-quality research, and disputes over power and financial resources. Another study recognized like factors as obstructions, plus the organizational system of government and its rigidity toward alteration, and the lake of a ‘functioning policy network’ including the policy-maker, researcher, and official [25-28].

Certainly, the most frequently presented associated with the absence of particular techniques or skills, knowledge, and education or training. This is confirmed by several studies, interestingly, these barriers were parallel to the facilitators. The health professionals showed that certified education is as the facilitator for barriers including the absence of particular techniques or skills, knowledge, and education or training. Most of research reported that knowledge achieves during education and autonomous learning. This is coordinated with previous studies that representative formal education is deficient in health professional training programs [25-29]. For example, a study of training in sleep recommended several negative results related to the absence of training in sleep, counting unsuitable interferences being delivered, unsuitable or referrals to specialists, non-evidence-based practice, or the absence of consideration to sleep symptoms [30-32]. The lack of skills, knowledge, education, techniques, and training presented to be mainly salient for nurses, and one barrier at least associated with this issue. There is obviously a requirement to improved knowledge of how to make possible education between health professionals to make certain that high-quality patient care [24-29]. Time is one of the most frequently presented barriers to EBP. In particular, the lack of time repotted as the barriers by more one-half of general practitioners who responded to questions. Interestingly, although the absence of time is one of the most frequently reported barriers to EBP, time was identified in a few studies as facilitators. Whereas, it is surely the case that health professionals have a lot of demands on their time in both practice and training [10,13,33,34]. The outcomes of studies were identified as the employ of the internet as the highest-rated skill for increasing EBP quality. The local internet access is restricting in the workplace; therefore, internet access may not give access to the types of databases that include the vast body of investigation. Therefore, considering the results of the present study and the similar other studies, providing appropriate facilities for implementing EBP, providing sufficient time for study and evidence-based action through volume reduction work, increasing human resources, training in time management, providing training principles of research methodology, applying research results, training principles and standards of EBP and establishing legal, political and administrative infrastructures, implementation of research results by service providers with monitoring of professional, ethical and some legal aspects, providing strategies for enhancing physician collaboration, and conducting language courses seem inevitable for the development and success of EBP. In reviewing the scientific sources on EBP, it was found that reference books and the internet have the most use, which is in agreement with the results of Oliveri *et al*. [40] in Denmark. Among the four most widely used sources, articles and journals were the least used, while in the study of Krahn *et al*. [41] in Germany, articles and journals were the most frequently used sources of information. Due to the limited information provided in the reference books and the lack of up-to-date information [42], the use of articles and journals is recommended. Consideration the low rate of use of articles that may be due to a lack of reading proficiency due to lack of English proficiency, removing these and other potential barriers is essential for evidence-based practice. The results of the studies show the low level of knowledge, practice and using of evidence among health care providers [43-45], while in some studies, the level of knowledge, knowledge, practice and using EBP rate is greater [46-48]; increasing knowledge, attitude, and use of evidence in care by providing appropriate training, providing financial and non-financial incentives, appropriate culture building, and other measures are necessary in this regard and the authorities and policymakers in this field should pay more attention. Scientific and professional care, patient-centered care, and attention to the quality of service are key concepts in evidence-based practice [49-51]. The results of interventional studies in EBP have shown that they have been effective in promoting EBP, and the results of recent studies and interventions have also improved EBP [52-56]; therefore, consideration to the small number of interventional studies, and the positive effects of interventions; the designing and implementing efficient interventions to improve EBP can be an effective solution. Weaknesses of the present study include failure to review abstracts of papers published in congresses, and organizational reports, as well as failure to perform statistical analyzes such as meta-analysis of studies. Other weaknesses of this study are the limitations of access to some databases; however, the results of this study can be of the great application by examining the relatively different aspects of evidence-based performance that have been studied separately and in one-dimensional previous studies.

## Conclusion

 Generally, the results showed health service administrators should first identify barriers of EBP then transferred them to facilitators to the implementation of proper and efficient EBP.

## Acknowledgment

 The authors would like to express their appreciation to the reviewers to evaluated articles.

## Conflict of Interest

 The authors declare that they have no conflicts of interest.

**Table 1:** Studies’ Characteristics

**Author’s Name**	**Sample**	**Design**	**Purpose**	**Barriers**	**Facilitators**	**Quality**
Gifford *et al*. (2018) [1]	Staff nurses, head nurses and directors (n=13)	Descriptive qualitative	Evaluated facilitators and barriers of EBP in the Hunan, a low grown province in China	A shortage of accessible evidence in Chinese, nurses’ absence of knowledge of what EBP indicates, and worry that patients will be offended of obtaining overhaul that is regarded as modern.	Administration support and the fast grown of social network services	Fair
Duncombe (2018) [2]	100 nurses	Descriptive, comparative study, they obtained data by self-administered questionnaires.	Explored barriers and facilitators of evidence-based practice in the Bahamas, in the hospital and community setting related to geriatric, psychiatric	“Inadequate resources for implementing research findings and “Inadequate training in research methods”	“Training in research methods” and “Organizational policies and protocols that are evidence-based”	-
Sullivan *et al*. (2017) [11]	14 pediatric surgeons	Qualitative -semi-structured interviews	Assessed facilitators and barriers to the perforation of EBP among pediatric surgeons	Resource limitations and time constraints, a lack of required skills, the commonly poor quality of data in pediatric surgery, and a belief that remains to trust on a training style of education.	Having a native peer/champion that supports EBP employment, and working in a research hospital	good
Oh *et al*. (2016) [4]	73 academic faculty nurses from 54 universities	Mixed method	Investigated self-efficacy, course needs, barriers, and facilitators related to the nurses	Absence of initial investment, skill, and knowledge for teaching EBP; rules-oriented nursing culture, hierarchical; limited research application and dissemination, latent student overloads in treating EBP	The collaboration in hospitals and schools; the importance of EBP to the work of nursing; and ongoing teaching in teaching/utilizing EBP	Fair
Majdzadeh *et al*. (2012) [5]	Policy-makers and managers of the Ministry of Health and Medical Education (thirteen in-depth interviews and six focus group discussions)	Qualitative in-depth interview	Evaluation of the obstructions to EBDM in Iran’s health system	They reported that important barriers to EBDM including standards for choosing decision-makers, organizational values, and the approach to near EBDM.	-	Fair
Jacobs *et al*. (2010) [6]	447 of chronic disease practitioners at the regional and state levels, the involvement of the NACDD	Descriptive qualitative	Evaluation of chronic disease practitioners’ self-reported barriers to EBDM	They reported that organizational barriers have higher scores than personal barriers. The main informed obstructions to EBDM were inadequate funding, absence of rewards/incentives, an acuity of state legislators not supportive evidence-based policies and interventions, and sense the require to be a professional on various subjects	-	Fair
Pagoto *et al*. (2007) [7]	37 behavioral professionals	Qualitative study	Evaluation and describe the main barriers and facilitators to evidence-based practice (EBP).	The professionals reported that lack of training and negative attitudes about EBP are most frequently barriers.	They showed that a growing evidence-base is a major facilitator.	Fair
Chang *et al*. (2010) [8]	89 Taiwanese nursing homes	Quantitative, descriptive study	Investigation of perceived facilitators and barriers, and also attitudes toward to research utilization between 89 Taiwanese RNs	The most common barriers were including difficulty understanding statistical analyses, inadequate authority to change practice, and supposed isolation from informed coworkers with whom to discuss the investigation	The facilitators were efficient research training, accessibility to Internet facilities and improved computers, and collaboration with academic nurses	-
Yadav *et al*. (2012) [9]	Sample size was 370 of psychiatric nurses	A descriptive cross-sectional study	Evaluation of the facilitators, barriers, and skills in developing EBP between psychiatric nurses in Ireland	The greatest barriers were insufficient resources to modify practice and insufficient time to find and read research reports.	The most supportive resource for altering was reported as practice development coordinators. They reported that the highest-rated skill was using the Internet to search, and the lowest-rated skill was using research to modify practice for developing evidence-based practice.	-
Boerner *et al*. (2015) [10]	124 Canadian health professionals	Descriptive qualitative	Evaluation of evaluated facilitators and barriers of the evidence-based behavioral sleep-related car by 124 Canadian health professionals	The main barrier and facilitator associated with training, knowledge, and education.	The facilitators included beliefs and supportive sleep attitudes, and the barriers included require to institutional support and time.	Fair
Kaper *et al*. (2015) [12]	537 international expert clinicians.	Descriptive qualitative	Evaluation of facilitators and barriers for EBP in patient care	They reported the EBP inventory consists of 26 items in 5 scopes: subjective norm, decision making, perceived behavior control, attitude, and behavior and intention	-	Fair
Malik et al. (2016) [13]	135 nurses employed in a tertiary health care network in Victoria, Australia.	Descriptive qualitative	Evaluation of the factors related to EBP	They showed that the absence of skills and knowledge, restricted support, poor time allowance, and inadequate resources are as barriers to acceptance of EBP in health care.	The adequate resources, governmental support, and admittance to long-term education are as factors supporting of acceptance of EBP	Fair
